# Identification of Myeloid Derived Suppressor Cells in Dogs with Naturally Occurring Cancer

**DOI:** 10.1371/journal.pone.0033274

**Published:** 2012-03-13

**Authors:** Michelle R. Goulart, G. Elizabeth Pluhar, John R. Ohlfest

**Affiliations:** 1 Department of Veterinary Clinical Sciences, University of Minnesota, Saint Paul, Minnesota, United States; 2 Department of Pediatrics, University of Minnesota, Minneapolis, Minnesota, United States of America; 3 Department of Neurosurgery, University of Minnesota, Minneapolis, Minnesota, United States of America; King's College London, United Kingdom

## Abstract

Dogs with naturally occurring cancer represent an important large animal model for drug development and testing novel immunotherapies. However, poorly defined immunophenotypes of canine leukocytes have limited the study of tumor immunology in dogs. The accumulation of myeloid derived suppressor cells (MDSCs) is known to be a key mechanism of immune suppression in tumor-bearing mice and in human patients. We sought to identify MDSCs in the blood of dogs with cancer. Peripheral blood mononuclear cells (PBMCs) from dogs with advanced or early stage cancer and from age-matched healthy controls were analyzed by flow cytometry and microscopy. Suppressive function was tested in T cell proliferation and cytokine elaboration assays. Semi-quantitative RT-PCR was used to identify potential mechanisms responsible for immunosuppression. PBMCs from dogs with advanced or metastatic cancer exhibited a significantly higher percentage of CD11b^+^CD14^−^MHCII^−^ cells compared to dogs diagnosed with early stage non-metastatic tumors and healthy dogs. These CD11b^+^ CD14^−^MHCII^−^ cells constitute a subpopulation of activated granulocytes that co-purify with PBMCs, display polymorphonuclear granulocyte morphology, and demonstrate a potent ability to suppress proliferation and IFN-γ production in T cells from normal and tumor-bearing donors. Furthermore, these cells expressed hallmark suppressive factors of human MDSC including ARG1, iNOS2, TGF-β and IL-10. In summary our data demonstrate that MDSCs accumulate in the blood of dogs with advanced cancer and can be measured using this three-marker immunophenotype, thereby enabling prospective studies that can monitor MDSC burden.

## Introduction

Cancer is the leading cause of death in adult dogs in the United States, Australia, Japan and Europe and is considered the major health care concern of pet owners. Approximately four million dogs are diagnosed with cancer each year in the United States [1]. Naturally occurring malignances in dogs share many features with human cancers including similar tumor biology, genetics, incidence rates, histological appearance, and response to conventional treatments (reviewed in [2]). Tumors in dogs progress relatively faster than the same disease in humans, allowing questions related to treatment efficacy (progression and survival) to be addressed more rapidly in dogs. An important advantage of the dog model is the ability to test experimental therapeutics at human scale doses in the setting of minimal residual disease, which is difficult to do in a meaningful way in small rodents that have relatively rapid tumor growth kinetics. In addition, because the standard of care for most canine tumors is poorly established, there is much more flexibility in study design compared to human clinical trials. Collectively these features make the dog an outstanding platform for translational medicine.

Pet dogs with cancer are rapidly becoming an important tool used in drug development. One of the best examples of this is the recent parallel development of SU11654, a multi-targeted tyrosine kinase inhibitor, and sunitinib malate (SU11248). Both drugs are potent inhibitors of PDGFR, VEGFR, KIT, and FLT3. Studies in dogs with various solid tumors revealed that plasma concentration of SU11654, the mutational status of KIT, and the inhibition of KIT phosphorylation were strongly predictive of clinical efficacy. Optimal dosing parameters and toxicity were established in dogs as well. These pioneering studies greatly facilitated the further development of this entire class of drugs, most notably the approval of sunitinib malate by the U.S. Food and Drug Administration for the treatment of renal cell carcinoma (RCC) and gastrointestinal stromal cell tumors, which often contain similar KIT mutations [3]. It was later recognized that sunitinib markedly depletes MDSCs and restores T cell function in human RCC patients [4], an observation that could not have been made in dogs at the time because of limited canine reagents and poorly defined markers for canine leukocytes. We, and others, are testing novel immune-based therapies in dogs with various malignancies, but immune monitoring in these studies has been confounded by the same problem. To put the field in perspective, a surface immunophenotype for canine natural killer cells has not been defined, the MHC alleles are poorly understood, and many of the markers used rely on cross-reactive antibodies whereby specificity must be tested empirically. It is crucial that new reagents are developed and that the immunophenotypes of all major canine leukocytes subsets are determined. Laying this basic foundation will allow unique insights to be made as new small molecule drugs and immunotherapies are tested in dogs as a prelude to human trials.

The accumulation of MDSCs in tumor-bearing mice and humans with cancer is known to be a key mechanism of tumor escape from immune surveillance [5], [6], [7]. MDSCs comprise a phenotypically heterogeneous population of myeloid cells in early stages of differentiation that expand in cancer and many other pathological conditions, and have a potent ability to suppress T cell function, especially T cell proliferation and effector cytokine production [6], [8]. MDSCs may be divided into monocytic and granulocytic subtypes. One source of controversy in this field is that MDSC heterogeneity has made comparisons between cancer patients and murine tumor models challenging (see reference [9] for excellent perspective). The molecular mechanisms by which MDSCs inhibit T cell function are under investigation. Studies have implicated up-regulation of arginase 1 (ARG1), inducible nitric oxide synthase (iNOS2) and reactive oxygen species (ROS) as important factors for MDSC-mediated immune suppression [8], [10], [11]. ARG1 can profoundly impair T cell function at the tumor site by L-arginine depletion, triggering the amino acid starvation response and apoptosis in lymphocytes [7]. Another mechanism of immune suppression is chemokine nitration, which blunts effector T cell infiltration into the tumor site [12]. Furthermore, MDSC expansion is associated with downregulation of L-Selectin on CD4^+^ and CD8^+^ T cells [13]. This reduces T cell trafficking to secondary lymphoid organs where tumor-reactive T cells can be primed [13]. Due to the ability of MDSCs to downregulate the immune response against tumors in mice and in humans, we hypothesized that these cells would also play an important role in tumor-induced immune suppression in dogs with cancer. Hence, the objective of this study was to identify surface markers that characterize the existence of MDSCs in dogs.

## Materials and Methods

### Study Population and sample collection

The description of all dogs in this study is summarized in **Tables 1** and **2**, with further detail provided in **Tables S1** and **S2**.

**Table 1:** Characteristics of dogs with cancer in the study.

Age (yrs) - Mean (Range)	9 (2–14)
**Gender**	
Male/Neutered	22
Male/Intact	2
Female/Spayed	21
**Processed Samples**	
Fresh	21
Frozen	24
**Breed**	
Labrador Retriever	12
Mixed Breed	5
Golden Retriever	3
Greyhound	2
Boxer	2
Border Collie	2
Beagle	2
Scottish Terrier	1
Bull Mastiff	1
Rottweiler	1
Dalmatian	1
Great Dane	1
Bernese Mountain Dog	1
German Wirehaired Pointer	1
German Shepherd Dog	1
West Highland White Terrier	1
Gordon Setter	1
Weimaraner	1
Rhodesian Ridgeback	1
Rat Terrier	1
Newfoundland	1
Miniature Poodle	1
Chow Chow	1
English Springer Spaniel	1
Total	45

**Table 2:** Characteristics of healthy dogs in the study.

Age (yrs) - Mean (Range)	8 (2–13)
**Gender**	
Male/Neutered	7
Male/Intact	1
Female/Intact	2
Female/Spayed	8
**Processed Samples**	
Fresh	6
Frozen	12
**Breed**	
Labrador Retriever	4
Golden Retriever	2
English Setter	1
Shih Tzu	2
Mixed Breed	2
German Shepherd dog	1
German Wirehaired Pointer	1
Red Tick Hound	1
Poodle	1
Cocker Spaniel	1
Catahoula Hound mix	1
Greyhound	1
Total	18

**Table S3** is a summary of samples assayed in each figure. Clinical data were obtained from medical records. Control dogs were determined to be healthy based on physical examination, owner observations and complete blood count exams. For dogs with cancer, the diagnosis and tumor staging were based on complete physical examinations, histopathology of tumor biopsy specimens, blood work and specialized imaging tests, such as CT scans, ultrasound or radiographs, to assess tumor location and size, as well as the presence of metastatic disease. Dogs with large, necrotic or multiple masses, lytic or severe bone destruction (with osteosarcoma) or presence of metastasis, were placed into the advanced stage/metastatic group. Animals presenting with small masses or no metastatic nodules were placed into the early stage non-metastatic group. **Tables S1** and **S2** also list specifics about any treatment that dogs with cancer had received prior to or at the time of blood collection for this study.

Blood samples from both cancer and healthy control dogs were obtained specifically for this study. Samples were collected in heparinized tubes by the Oncology and Community Practice Services of the Veterinary Medical Center at the University of Minnesota according to Institutional Animal Care and Use Committee guidelines. The samples were drawn after the owners signed the client consent form. The Institutional Animal Care and Use Committee (IACUC) reviewed and approved the study entitled as “Flow Cytometric Immunophenotyping of Peripheral Blood Cells in Dogs” via designated member review under the code number 0912A75493. Unless explicitly stated otherwise, the cells being analyzed for this manuscript co-purified with peripheral blood mononuclear cells (PBMCs) of dogs with cancer or age-matched healthy controls that were isolated using Ficoll (Sigma) gradient centrifugation as follows. Heparinized peripheral blood was diluted 1∶3 with sterile PBS (Invitrogen) and layered over Ficoll-Histopaque (Sigma). Samples were centrifuged at 400-× g for 30 min. The PBMCs collected at the interface were transferred to a fresh tube, washed twice with PBS, and resuspended with freezing solution consisting of 90% fetal bovine serum (Invitrogen) 10% Dimethyl sulfoxide (DMSO) (Sigma) and then frozen at −80°C. Lastly, PBMCs were thawed for 2 minutes in a 37°C water bath before staining and analysis. For analysis of fresh samples, PBMCs were isolated as above, resuspended in FACS buffer, stained with antibodies and immediately analyzed by flow cytometry or FACS as indicated.

### Flow Cytometric Analysis

PBMC samples were isolated from fresh blood or thawed and resuspended in FACS buffer. Nonspecific antibody binding was blocked by pretreatment of cells with 10 µg/mL canine gamma-globulin (Jackson Immunoresearch) for 20 min at room temperature. Cells were first labeled using indirect staining with 0.1 µg of unconjugated mouse anti-dog CD11b antibody (clone CA16.3E10, AbD Serotec) or IgG1 isotype control (AbD Serotec) and 0.5 µg of PE-conjugated goat F(ab′)2 anti-mouse IgG (Abcam) secondary antibody at 4°C for 30 min in a dark room. Following indirect staining, cells were washed twice and stained with 0.3 µg of FITC-conjugated rat anti-dog MHCII (clone YKIX334.2, AbD Serotec) and 0.15 µg of the cross-reactive, Alexa fluor 647-conjugated mouse anti-human CD14 antibody (clone TÜK4, AbD Serotec) or isotypes controls at 4°C for 30 min in a dark room according to manufacturer's protocol. Antibody-labeled cells were washed twice and re-suspended in FACS buffer. Cells were incubated for 10 minutes at room temperature in the dark with 7-amino-actinomycin D (7AAD, final concentration of 1 µg/mL; Calbiochem) and then analyzed on a Becton Dickinson Canto three-laser flow cytometer. Data were further analyzed with FlowJo software (Tree Star). Analysis gates were set based on the 7AAD negative population. The percentage of MDSCs was calculated based on the percentage of CD11b^+^CD14^−^MHCII^−^ cells within the overall live PBMC population. In one experiment (**Figure S1**), anti-mouse PE-conjugated CD11b (clone M1/70 eBioscience) and anti-mouse APC-conjugated Gr-1 (clone RB6-8C5 eBioscience) antibodies were also used to verify cross-reactivity with dog cells.

### Isolation of MDSCs, PMNs and T cells

For functional assays, RT-PCR and cell morphology analysis, fresh blood samples from a tumor-bearing dog were used for isolation of CD11b^+^CD14^−^MHCII^−^ or CD11b^+^CD14^+^MHCII^−^ cells, as indicated, using a BD FACSAria cell sorter. For T cell isolation, PBMCs were isolated as previously described from fresh blood samples of healthy dogs and stained with 0.3 µg of FITC-conjugated mouse anti-dog CD3 (clone CA17.2A12, AbD Serotec), 0.15 µg of Pacific blue-conjugated mouse anti-dog CD4 (clone YKIX302.9, AbD Serotec) and 0.15 µg of Alexa700-conjugated mouse anti-dog CD8 (clone YCATE55.9, AbD Serotec) antibodies. Polymorphonuclear leukocytes (PMN) were purified from the cell pellet of a Ficoll gradient from healthy dog blood samples, after removal of the PBMCs (at the top of gradient) and erythrocytes by RBC lysis buffer (eBioscience).

### *Ex Vivo* Proliferation

Analysis of MDSC inhibitory activity on T cell proliferation was measured by ^3^H-thymidine incorporation into DNA. Briefly, PBMCs from the indicated dogs were seeded into U-bottom 96-well plates (5×10^4^cells/well) in medium consisting of RPMI 1640 containing L-arginine (150 µM) (Invitrogen) supplemented with penicillin/streptomycin (Invitrogen) and 10% heat-inactivated fetal bovine serum (Invitrogen) at 37°C, in a 5% CO_2_ incubator. CD11b^+^CD14^−^MHCII^−^ or CD11b^+^ CD14^+^ MHCII^−^ cells from a dog with cancer were sorted and added to cancer (autologous) or healthy responder PBMCs as indicated. Concanavalin A (5 µg/ml) (Sigma) and recombinant human IL-2 (10 IU/ml) (R&D systems) were used to stimulate T cell proliferation. Non-stimulated PBMCs were used as negative control. PBMCs or PMNs were co-cultured with healthy PBMCs to control for the effect of simply adding additional cells to the suppression assay as indicated. Plates were cultured for 72 h, then pulsed with 1 µCi of ^3^H-thymidine (Amersham Pharmacia Biotech) for 18 hrs at 37°C. Cells were harvested onto glass fiber filters (Perkin Elmer), washed, dried, and counted. Proliferative responses were measured by ^3^H-thymidine incorporation into the DNA using a Matrix 96 Direct Beta Counter (Packard). All experiments were performed in triplicate.

### IFN-γ Analyses

FACS-isolated CD11b^+^CD14^−^MHCII^−^ cells from a cancer dog were co-cultured with PBMCs isolated from a healthy dog using the same method as the proliferation assay. After 72 hrs of incubation the cell culture supernatants were collected and measured using a Quantikine canine IFN-γ ELISA kit according to the manufacture's instructions (R&D systems). Samples were assayed colorimetrically, in triplicate, using a Microplate Reader Synergy2 (Biotek) and analyzed with Microplate Data Collection and Analysis Software Gen5 (Biotek).

### Cytospin

FACS-isolated CD11b^+^CD14^−^MHCII^−^ cells were stained using a modified Giemsa stain (Diff-quick, Astral Diagnostics Inc) for cell morphology evaluation and observed using a DME microscope (Leica) at 63× power magnification. Pictures were acquired with an EC3 camera (Leica).

### RNA extraction and RT-PCR

RNA was extracted from FACS-isolated CD11b^+^CD14^−^MHCII^−^ cells or healthy dog PMNs, using an RNAeasy plus Mini kit (QIAGEN) according to the manufacturer's protocol. RNA concentrations were evaluated using a ND (100) spectrophotometer (Nanodrop). To detect expression of ARG1 and iNOS2 enzymes, gene-specific primers were designed based on the canine ARG1 and iNOS2 sequence; primer sequences for housekeeping gene were designed from canine β-actin gene using Primer3Plus (http://www.bioinformatics.nl/cgi-bin/primer3plus/primer3plus.cgi). For detection of cytokines IL-10 and TGF-β, primer sequences of IL-10 and TGF-β were obtained from published sources [14]. The BLAST algorithm (http://blast.ncbi.nlm.nih.gov/Blast.cgi) was used to ensure primer specificity to the target gene. First strand cDNA synthesis was done using a QuantiTect Reverse Transcription kit (QIAGEN). The two-step PCR reaction was carried out in a 12.5-µl volume containing 2× SYBR green master mix (Quanta Biosciences), 0.675U GoTaq Polymerase, 2 nM MgCl_2_ (Promega), 0.2 mM dNTPs (Stratagene), 0.2 µM of each primer pair and 50 ng of cDNA template. Reaction conditions consisted of initial denaturation at 94°C for 2 min, then cycles of denaturation at 94°C for 30 s, annealing at 60°C for 45 s, elongation at 72°C for 45 s and final elongation at 72°C for 5 min in a DNA Engine Thermal Cycler (Bio-rad). The optimum annealing temperature for each primer pair was established prior to the study (see primer sequences in **Table S4**). PCR products were run on 2% agarose gels containing 0.5 µl/ml ethidium bromide and imaged under 590 nm ultra-violet light on a Eagle Eye II image station (Stratagene). Negative control reactions were performed using RNA that was not subjected to reverse transcription PCR.

### Statistical Analysis

The differences between two groups were analyzed using unpaired, two-tailed Student's *t test*. All tests were performed with Prism 4 software (Graph Pad Software, Inc). P values <0.05 were considered to be statistically significant.

## Results

### Dogs with advanced cancer have elevated levels of granulocytic CD11b^+^CD14^−^MHCII^−^ cells that co-purify with PBMCs

**Figure 1:**
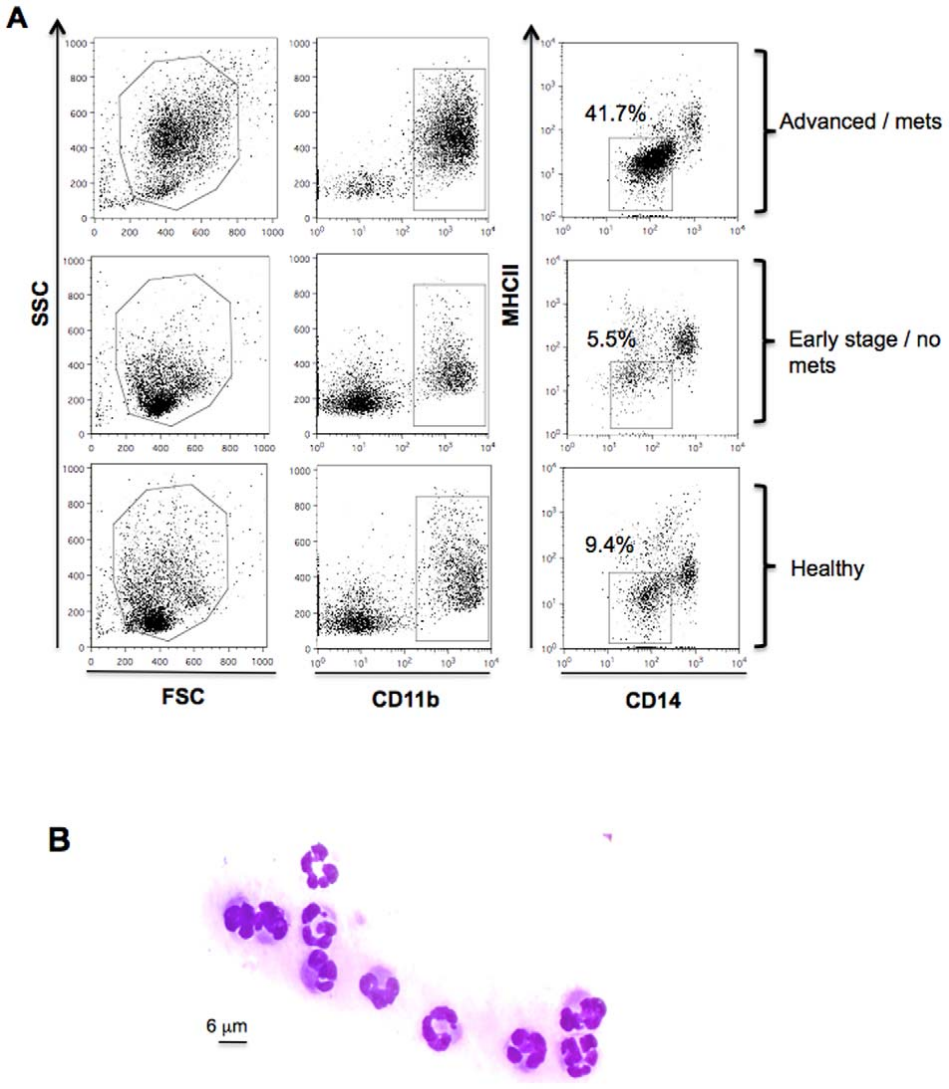
Immunophenotyping gating strategy and morphological analysis for MDSC identification in peripheral blood of dogs. PBMCs from healthy dogs and dogs with cancer were stained for the myeloid marker CD11b, monocytic marker CD14 and MHC II. (A) Representative flow cytometric analysis of forward and side scatter and gated CD11b^+^CD14^−^MHCII^−^ cells from dogs with advanced or metastatic tumors compared to dogs with early stage non-metastatic tumors and healthy control dogs. Plots are representative of dog with advanced metastatic hemangiosarcoma (top), early stage bladder transitional cell carcinoma (middle) and a healthy dog. (B) FACS sorted CD11b^+^CD14^−^MHCII^−^ cells were stained with diff-quick for cell morphology evaluation. A representative example of polymorphonuclear granulocyte morphology of CD11b^+^CD14^−^MHCII^−^ cells is shown at 63× magnification.

Peripheral blood samples from 45 dogs diagnosed with cancer and 18 healthy control dogs were collected (**Tables 1** and **2**). All dogs with cancer underwent clinical staging of their disease by performing complete physical examinations, blood work, imaging to assess tumor location and size and metastases, and histopathological diagnosis made from diagnostic aspirate or biopsy of the tumor. Among the 45 dogs diagnosed with cancer, 30 dogs were classified as having advanced or metastatic disease and 15 dogs were classified as early stage/non-metastatic or low grade disease based on clinical staging. Each group was further subdivided according to histological diagnosis into sarcomas, carcinomas or mast cell tumors (detailed in **Tables S1** and **S2**). The percentages of putative MDSCs in dogs with cancer and healthy dogs were evaluated by flow cytometry. PBMCs from dogs with advanced or metastatic cancer showed a marked increase in the CD11b^+^CD14^−^MHCII^−^ fraction of cells, which accounted for the majority of the cells in the live cell gate, compared to dogs diagnosed with early stage non-metastatic tumors or healthy dog controls (**Fig. 1A**). This subset of cells exhibited a polymorphonuclear granulocytic morphology at heterogeneous stages of development (**Fig. 1B**), which resembles a granulocytic subset of MDSCs identified in mice [15] and humans [16].

**Figure 2:**
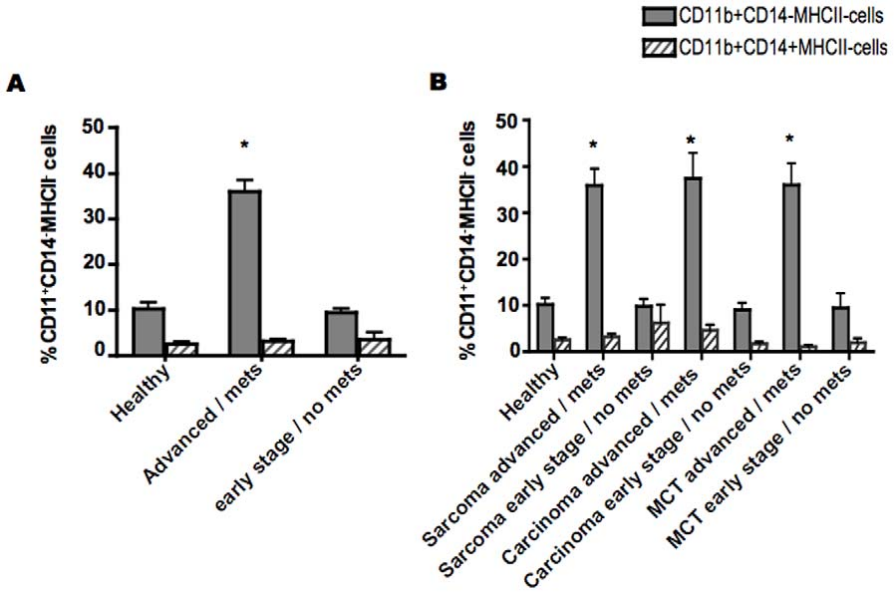
Percentages of circulating CD11b^+^CD14^−^MHCII^−^ cells in dogs with correlates with clinical tumor stage. (A) Analysis of average CD11^+^CD14^−^MHCII^−^ population frequency in dogs with advanced stage or metastatic tumors (n = 30) compared with early stage non-metastatic tumors (n = 15) and control dogs (n = 18). There was a significantly higher percentage of CD11b^+^CD14^−^MHCII^−^ cells in dogs with advanced cancer versus early stage non-metastatic tumors and healthy dogs (36.04% vs. 9.40% and10.24%, respectively. B) Average CD11b^+^CD14^−^MHCII^−^ population frequency in the major cancer subtypes: advanced stage or metastatic sarcomas (n = 18), early stage non-metastatic sarcomas (n = 6), advanced stage or metastatic carcinomas (n = 7) early stage non-metastatic carcinomas (n = 7), advanced stage or metastatic mast cell tumors (n = 5) and early stage non-metastatic mast cell tumors (n = 2) compared with control dogs (n = 18). Significantly elevated percentages were detected in all advanced tumors subtypes relative to early stage tumors and healthy dogs. Percentages of CD11b^+^CD14^+^MHCII^−^ cells were not significant between groups (* indicates P<0.001). Mean ± SEM are shown.

Dogs with advanced or metastatic cancer had a significantly greater fraction of putative MDSCs (36.04±2.542, mean ± SEM) compared to dogs with early stage non metastatic tumors (9.40±0.953, mean ± SEM) and healthy control dogs (10.24±1.412, mean ± SEM) (**Fig. 2A**). Moreover, this elevation in the CD11b^+^CD14^−^MHCII^−^ fraction did not appear to be restricted to a specific tumor type. The differences were statistically significant in dogs with sarcomas, carcinomas, and mast cell tumors compared with healthy controls (**Fig. 2B**). Conversely, the percentage of CD11b^+^MHCII^−^ cells that did express CD14 was not significantly different among any group. Therefore, the frequency of CD11b^+^CD14^−^MHCII^−^ cells that co-purify PBMCs correlates with tumor burden. This finding is in agreement with previously published data regarding MDSC levels and tumor burden in mice and humans [17], [18].

### CD11b^+^CD14^−^MHCII^−^ cells are functionally defined as MDSCs

**Figure 3:**
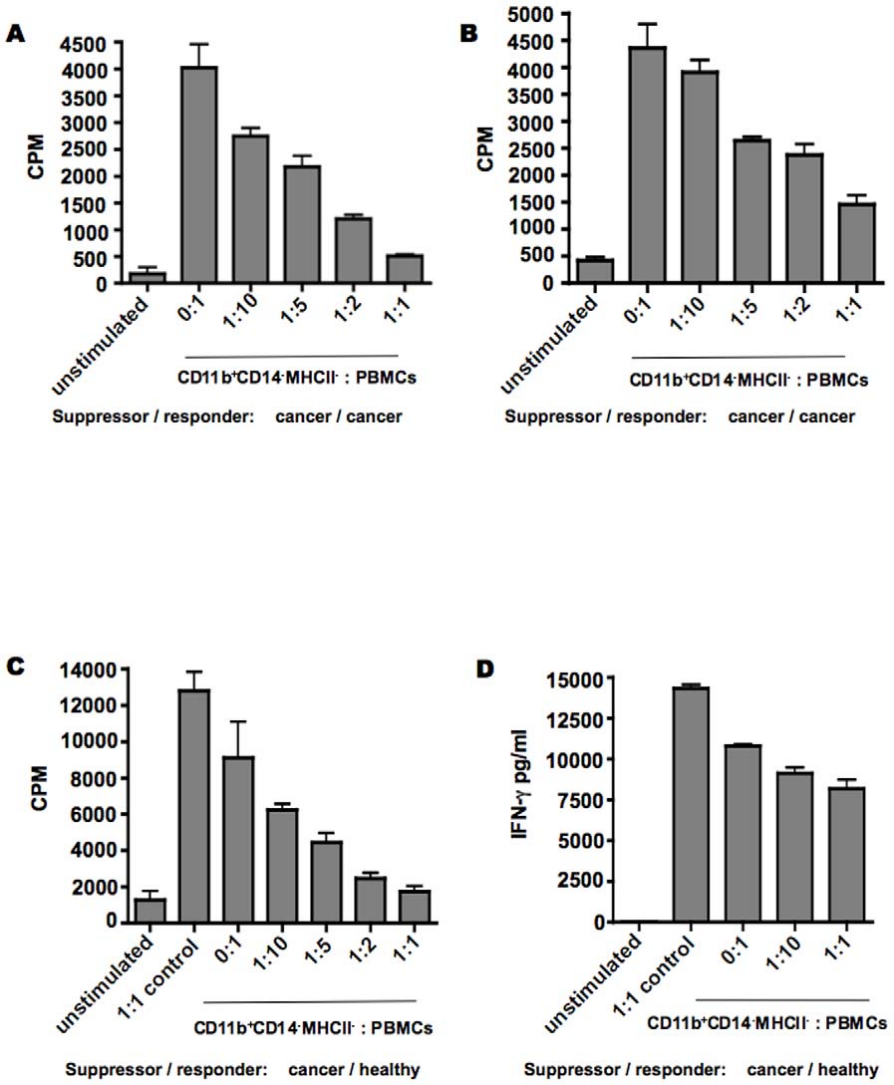
CD11b^+^CD14^−^MHCII^−^ cells suppress T cell proliferation and cytokine elaboration. CD11b^+^CD14^−^MHCII^−^ cells were sorted from peripheral blood sample of dogs with cancer and then co-cultured with autologous PBMCs (A, B) or healthy dog PBMCs (C) in the presence of mitogen for 72 hs. Representative examples from a total of eight dogs are shown. The graphs represent proliferative responses after addition of CD11b^+^CD14^−^MHCII^−^ isolated from a single dog with squamous cell carcinoma (3A), prostatic adenocarcinoma (3B) and osteosarcoma (3C). Non-stimulated PBMCs were used as negative control and PBMCs stimulated in absence of CD11b^+^CD14^−^MHCII^−^ cells were used as positive control for proliferation. PBMCs were also co-incubated with PMNs, to control for presence of additional cells (3C, 3D). Proliferative responses were measured by ^3^H-thymidine incorporation. CPM, counts per minute. Amount of IFN-γ secretion in the co-culture was determined using canine specific IFN-γ ELISA assay (3D). All experiments were performed in triplicate. Mean ± SEM are shown.

To test whether the CD11b^+^CD14^−^MHCII^−^ subset was able to inhibit T cell function, we conducted a series of co-culture experiments. Purified CD11b^+^CD14^−^MHCII^−^ cells from three different subtypes of cancer were co-cultured with autologous or healthy responder PBMCs. In all cases, CD11b^+^CD14^−^MHCII^−^ cells exhibited a potent ability to suppress proliferative responses in a dose-dependent manner. Representative examples of proliferative suppression are shown using samples from a dog with tonsillar squamous cell carcinoma (**Fig. 3A**) and prostatic adenocarcinoma (**Fig. 3B**). In order to determine if suppression was an artifact of using responders from tumor-bearing dogs, we assayed for proliferative suppression using normal responders. The addition of CD11b^+^CD14^−^MHCII^−^ cells, but not normal PMNs, impaired the proliferation of PBMCs from healthy dogs (**Fig. 3C**). Moreover, the amount of IFN-γ secretion was assessed in the conditioned medium from these co-cultures, revealing that CD11b^+^CD14^−^MHCII^−^ cells, but not normal PMNs, suppressed the secretion of IFN-γ (**Fig. 3D**).

### MDSCs suppress both CD4^+^ and CD8^+^ T cells

**Figure 4:**
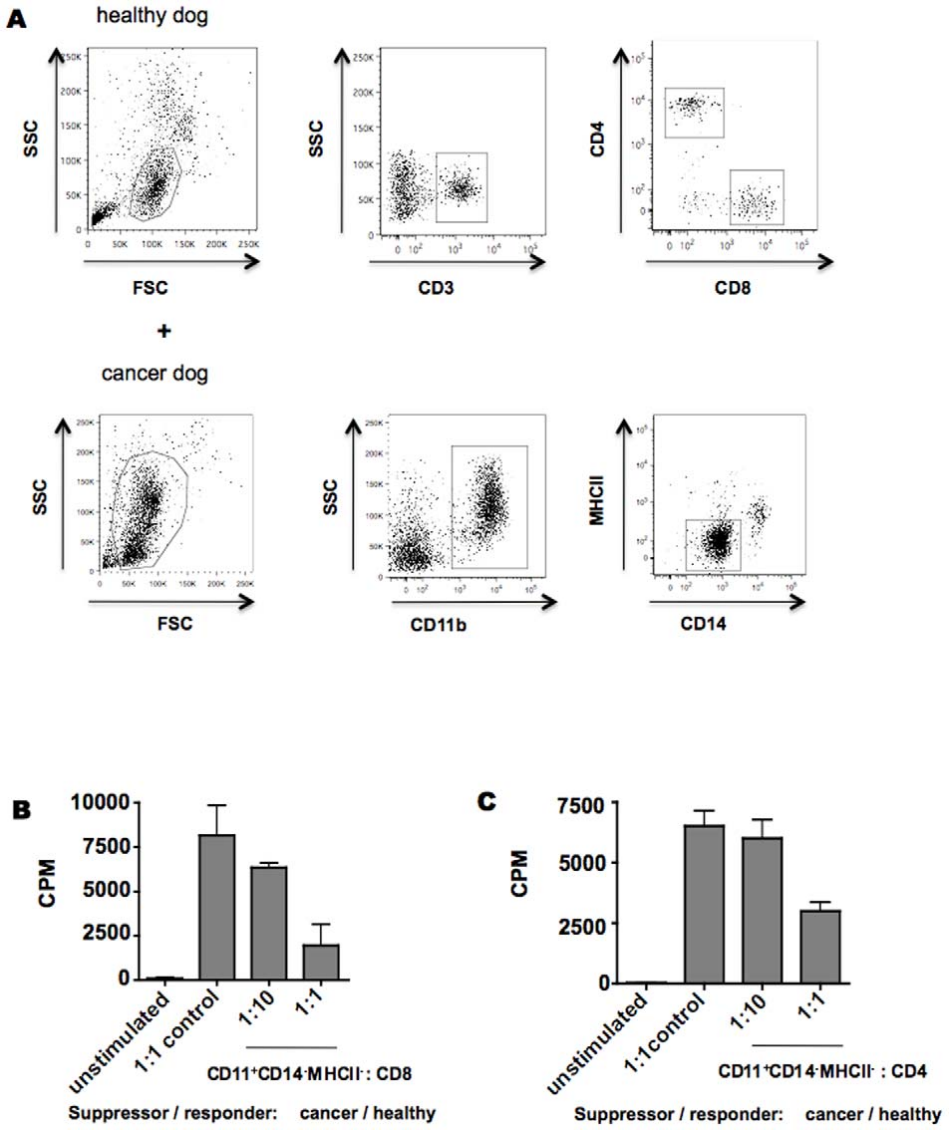
CD11b^+^CD14^−^MHCII^−^ cells suppress T cell proliferation. Facs sorted CD11b^+^CD14^−^MHCII^−^ cells isolated from a dog with osteosarcoma or healthy PBMCs were co-incubated with mitogen-stimulated CD4^+^ and CD8^+^ T cells isolated from a healthy dog for 72 hs. No stimulated cells were used as negative control. Proliferative responses were measured by ^3^H-thymidine incorporation from experiments performed in triplicate. CPM, counts per minute. Mean ± SEM are shown.

To further interrogate the direct effect on T lymphocytes, purified CD11b^+^CD14^−^MHCII^−^ cells from a dog with osteosarcoma were co-cultured with purified CD4^+^ and CD8^+^ T cells from a healthy dog for 72 h. Non-stimulated cells and CD4^+^ and CD8^+^ cells co-incubated with healthy PBMCs were used as controls. As expected, CD11b^+^CD14^−^MHCII^−^ cells inhibited the proliferation of CD8^+^ (**Fig. 4A**) and CD4^+^ T cells (**Fig. 4B**) while PBMCs from a normal dog did not. Taken together, these data demonstrate that CD11b^+^CD14^−^MHCII^−^ cells are indeed functionally defined as canine MDSCs.

### CD11b^+^CD14^−^MHCII^−^ cells express hallmark MDSC-derived immunosuppressive factors

**Figure 5:**
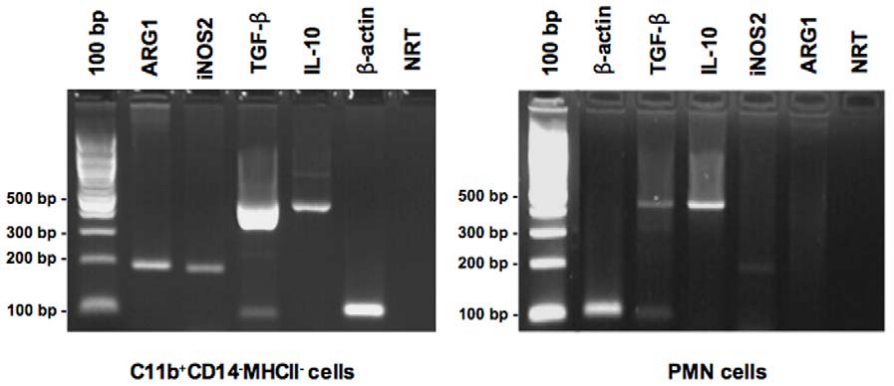
CD11b^+^CD14^−^MHCII^−^ cells express MDSC-derived immunosuppressive factors. RT-PCR analysis of FACS purified CD11b^+^CD14^−^MHCII^−^ cells detected expression of ARG1 and iNOS2, as well TGF-β and IL-10 immunosuppressive cytokines. ARG-1 expression was not detected in normal PMNs. CD11b^+^CD14^−^MHCII^−^ cells were isolated from the peripheral blood of a dog with osteosarcoma and PMNs were isolated from a healthy dog. NRT, RNA template in the absence of reverse transcriptase. Results are representative three experiments.

It has been shown that MDSCs can inhibit T cell function by the production of soluble factors such as arginase-1, reactive oxygen species, nitric oxide and TGF-β (8–10). In order to assess whether CD11b^+^CD14^−^MHCII^−^ cells from dogs with cancer could possibly utilize these mechanisms to mediate T cell suppression, we evaluated the expression of ARG1 and iNOS2, as well as the immunosuppressive cytokines TGF-β and IL-10, within this cell population and from PMNs isolated from peripheral blood of healthy dogs. PCR analysis of RNA extracted from FACS isolated CD11b^+^CD14^−^MHCII^−^cells confirmed the expression of ARG-1, iNOS2 enzymes and immunosuppressive cytokines TGF-β and IL-10 mRNA (**Fig. 5A**). In contrast, normal dog PMNs did not express ARG1, although iNOS, TGF-β and IL-10 mRNA were detectable (**Fig. 5B**). Because mRNA for ARG-1, iNOS2, TGF- β and IL-10 were all found, we conclude that these factors could play a role in the inhibition of T cell proliferation and effector function. However, since PMNs isolated from healthy dogs did not express detectable ARG-1 mRNA or impair T cell function, suggesting that ARG-1 may be a tumor-induced mechanism that MDSCs could employ for T cell suppression. This finding was not unexpected and has been previously documented in human MDSC studies [19].

## Discussion

The field of comparative oncology shows great promise to advance the development of novel therapeutics for pet dogs and human patients alike. However, the paucity of reagents and poorly defined immunophenotype of canine leukocytes has restrained our ability to understand tumor immunology in dogs with naturally occurring cancer. Our data demonstrates the existence of MDSCs in the peripheral blood of dogs, which are elevated in all types of advanced or metastatic cancer analyzed compared to early stage non-metastatic cancer and healthy controls. With this basic foundation of knowledge in place, it will now be possible to prospectively monitor MDSC burden in dogs treated with experimental drugs and immunotherapy. The CD11b^+^CD14^−^MHCII^−^ cell population that we defined as MDSC co-purified with PBMCs, had polymorphonuclear granulocytic morphology, suppressed T cell proliferation and effector function, expressed hallmark suppressive factors of human MDSC, and positively correlated with tumor burden. Proliferation assays revealed relatively weak proliferation in PBMCs from tumor-bearing dogs (**Fig. 3A,B**) compared to normal responders (**Fig. 3C**) in the absence of exogenous MDSC. This likely reflects elevated levels of endogenous (not experimentally added) MDSCs and regulatory T cells in the PBMCs from dogs with cancer. Furthermore, it is crucial to note that a second subset of MDSC that is more monocytic in nature is widely appreciated in murine and human tumor immunology. We found no evidence for selective expansion of a CD14^+^ monocyte-like cell in the blood of dogs with cancer. However, CD11b^+^MHCII^−^ cells that were purified from dogs with advanced cancer that were also CD14^+^ potently inhibited T cell proliferation (**Figure S2**), revealing that although monocytic MDSC are not a dominant population in dogs with cancer, they are indeed present. This finding of preferential expansion of granulocytic MDSC is not surprising and is in agreement with similar studies carried out in murine tumor models [**15**]. Overall, our data are consistent with a global state of immune suppression in dogs with advanced cancer that is likely attributable to several mechanisms.

The practical deliverable of this study is a simple three marker surface immunophenotype that can be used to prospectively monitor MDSC burden in dogs. We have performed pilot studies to look for additional markers. Specific preliminary results that are worth noting are as follows. We have been unable to demonstrate successful staining using anti-human CD66b antibodies. CD66b is an activation marker expressed on some human MDSC [19]. The most widely used marker for MDSC in the mouse is Gr-1, and an antibody against mouse Gr-1 cross-reacts nicely with canine cells, as does anti-mouse CD11b (**Figure S1**). Further studies will be required to determine if canine cells that are identified by anti-mouse Gr-1 and CD11b antibodies are indeed MDSCs.

One potential limitation of this study that many of the samples we analyzed were frozen, the thawed before analysis, which could have influenced cell viability. However, freeze-thaw did not significantly affect cell viability of either granulocytic or monocytic MDSC (**Figure S3**). We consider this a positive finding because canine MDSCs could be frozen from multiple time points in future prospective studies, then thawed and analyzed simultaneously to limit batch to batch variability. A second limitation is that the RT-PCR analysis of immunosuppressive molecules was qualitative, was performed on a small number of dogs (**Table S3**), and was not a direct comparison to matched healthy cells. We were not able to obtain adequate viable CD11b^+^CD14^−^MHCII^−^ cells from healthy dogs by FACS to directly compare to the same population from dogs with cancer due to their low frequency and apparently high rate of cell death following FACS. For this reason, normal PMNs isolated by gradient centrifugation were used for comparison in our studies. Quantitative mechanistic studies should be conducted to dissect which of the candidate molecules studied herein mediate T cell suppression. Additionally, some of the dogs had received treatment for their cancer. This is relevant because MDSC levels in human cancer patients have been shown to be influenced by prior therapy. It is also known that tumor burden and inflammation significantly affect circulating MDSC levels. Studies in mice have shown that accumulation and suppressive activity of MDSCs are regulated by the inflammatory milieu [20]. Thus treatment, such as surgical excision of the tumor, chemotherapy, and nonsteroidal anti-inflammatory drug (NSAID) administration, can alter the levels of these cells in the peripheral blood. Evaluation of the medical records of dogs in our study revealed that many dogs received some therapy prior to blood sample collection, which could have affected the levels of MDSCs in these samples (see **Tables S1** and **S2**). However, **Figure S4** demonstrates that treatment of dogs with advanced cancer did not significantly alter MDSC burden relative to dogs that had not been previously treated. Therefore, our study provides evidence that expanded MDSCs are likely a robust, general feature of cancer in canines despite genetic heterogeneity and a range of previous treatments (or lack of previous treatment).

In summary, we have identified a granulocytic subset of cells with immunosuppressive function that are elevated in dogs with advanced cancer that can be characterized as MDSCs. Canine MDSCs may be a potential target for therapeutic interventions in dogs with cancer. Furthermore, the study of MDSCs in dogs treated with experimental therapies should reveal unique insights into what might be expected in human patients. This cross-species comparison provides an attractive opportunity to move the field of translational medicine forward.

## Supporting Information

Figure S1 **Mouse anti-CD11b and Gr-1 antibodies cross-react with canine samples.** Fresh PBMCs from healthy dog and cancer patients were isolated by Ficoll, stained with anti-mouse CD11b and anti-mouse Gr-1 antibodies.
(TIF)

Figure S2 **CD11b^+^CD14^+^MHCII^−^ cells demonstrate ability to suppressive T cell proliferation.** (A) CD11b^+^CD14^+^MHCII^−^ cells were sorted from peripheral blood sample of an osteosarcoma dog (B) and co-cultured with healthy dog PBMCs in the presence of mitogen for 72 hs. Non-stimulated PBMCs were used as negative control and PBMCs co-cultured with healthy PMNs were used to control for the effect of adding cells to the assay. Proliferative responses were measured by ^3^H-thymidine incorporation. CPM, counts per minute. The experiment was performed in triplicate. Mean ± SEM are shown.
(TIF)

Figure S3 **Frequency of MDSCs measured was not significantly altered by cryopreservation.** MDSC percentages in fresh and frozen samples were assessed for comparison. Mean ±SEM are shown.
(TIF)

Figure S4 **No significant effect of pretreatment on MDSC burden.** Analysis of the average CD11b^+^CD14^−^MHCII^−^ population frequency in treated (n = 17) or untreated dogs with advanced stage or metastatic tumors (n = 13) compared to control dogs (n = 18). There was a significantly higher percentage of CD11b^+^CD14^−^MHCII^−^ cells in dogs with advanced cancer treated or untreated compared to healthy dogs (32.69±3.24%, 40.42±3.86% vs. 10.24±1.412%, respectively). N.S., not statistically significant (there was no significant difference between samples that had been treated compared to those from untreated samples). Mean ± SEM are shown (* indicates P<0.0001).
(TIF)

Table S1 **Summary data for dogs with advanced stage or metastatic tumors.**
(DOC)

Table S2 **Summary data for dogs with early stage non-metastatic tumors.**
(DOC)

Table S3 **Table of cancer patient samples and the experiment in which the PBMCs were used.**
(DOC)

Table S4 **Primer sequences for genes evaluated by semi-quantitative PCR.**
(DOC)
