# Supplementary figures and images for: Identification of Myeloid Derived Suppressor Cells in Dogs with Naturally Occurring Cancer
Source: PLoS One. 2012 Mar 13;7(3):e33274. doi: 10.1371/journal.pone.0033274 (PMC3302813; doi:10.1371/journal.pone.0033274)

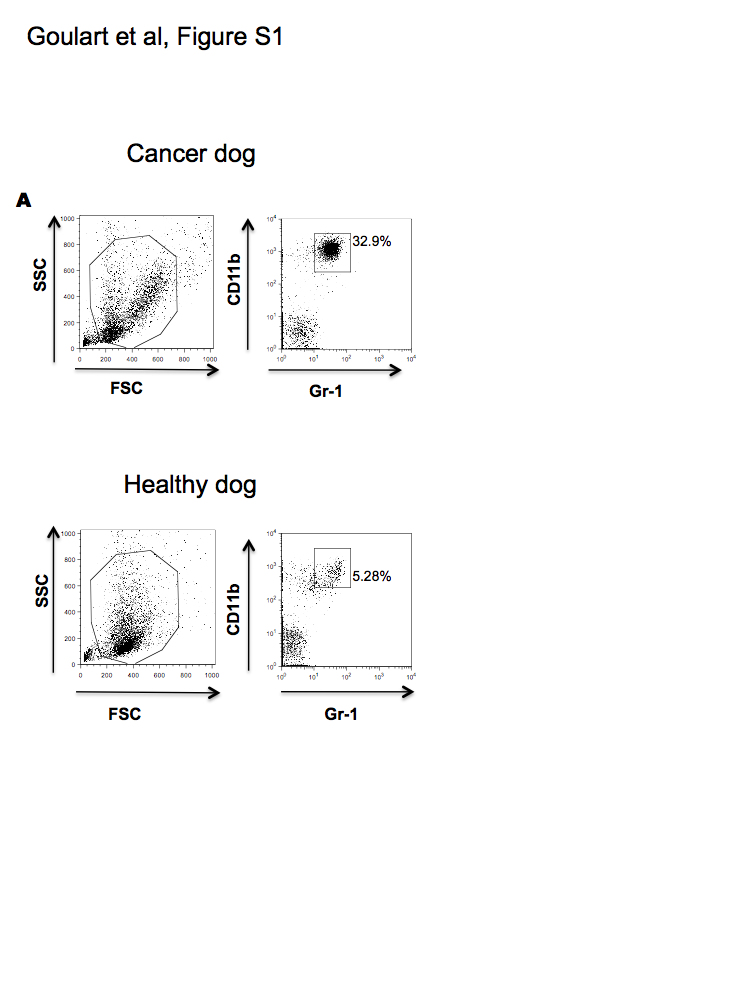

Supplement: Figure S1 — Mouse anti-CD11b and Gr-1 antibodies cross-react with canine samples. Fresh PBMCs from healthy dog and cancer patients were isolated by Ficoll, stained with anti-mouse CD11b and anti-mouse Gr-1 antibodies. (TIF) [file pone.0033274.s001.tif]

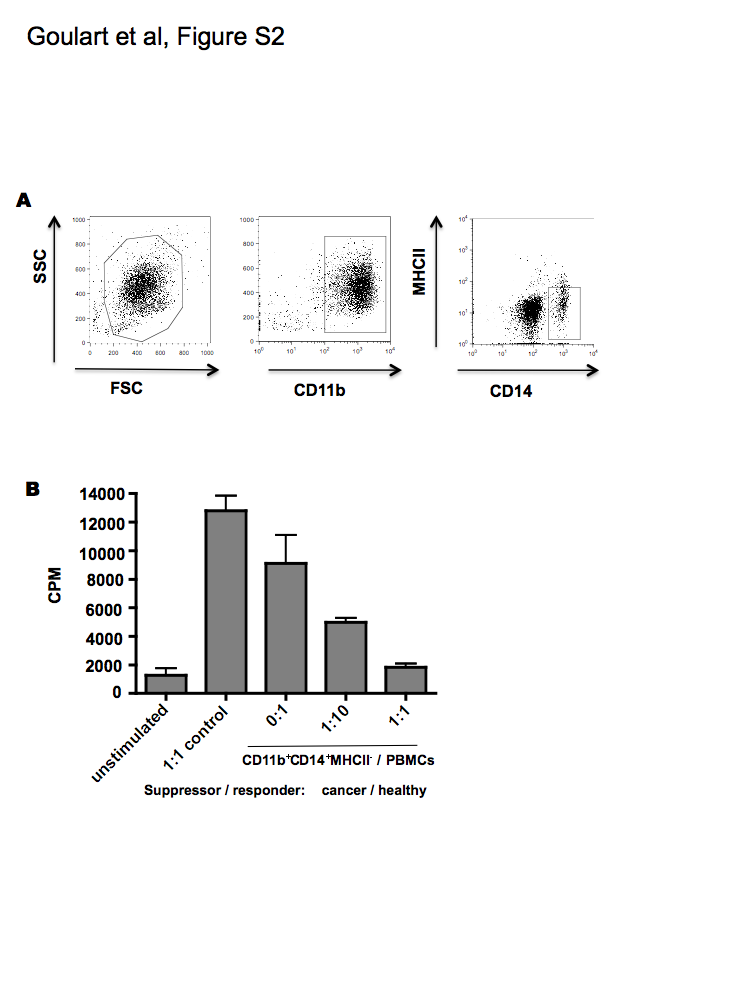

Supplement: Figure S2 — CD11b+CD14+MHCII− cells demonstrate ability to suppressive T cell proliferation. (A) CD11b+CD14+MHCII− cells were sorted from peripheral blood sample of an osteosarcoma dog (B) and co-cultured with healthy dog PBMCs in the presence of mitogen for 72 hs. Non-stimulated PBMCs were used as negative control and PBMCs co-cultured with healthy PMNs were used to control for the effect of adding cells to the assay. Proliferative responses were measured by 3H-thymidine incorporation. CPM, counts per minute. The experiment was performed in triplicate. Mean ± SEM are shown. (TIF) [file pone.0033274.s002.tif]

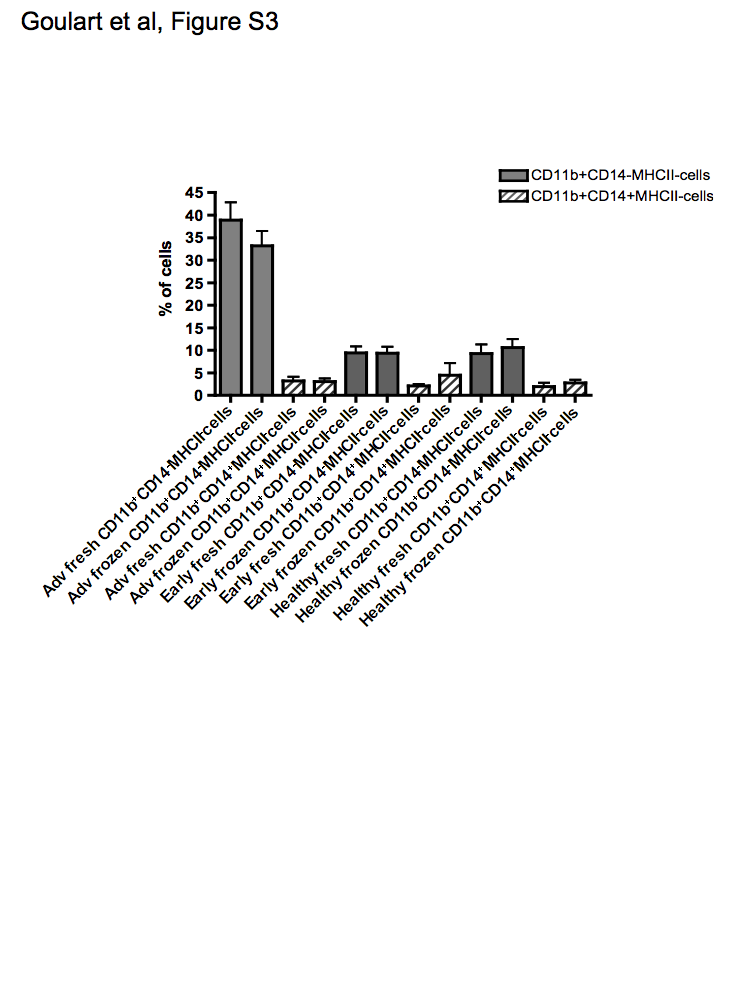

Supplement: Figure S3 — Frequency of MDSCs measured was not significantly altered by cryopreservation. MDSC percentages in fresh and frozen samples were assessed for comparison. Mean ±SEM are shown. (TIF) [file pone.0033274.s003.tif]

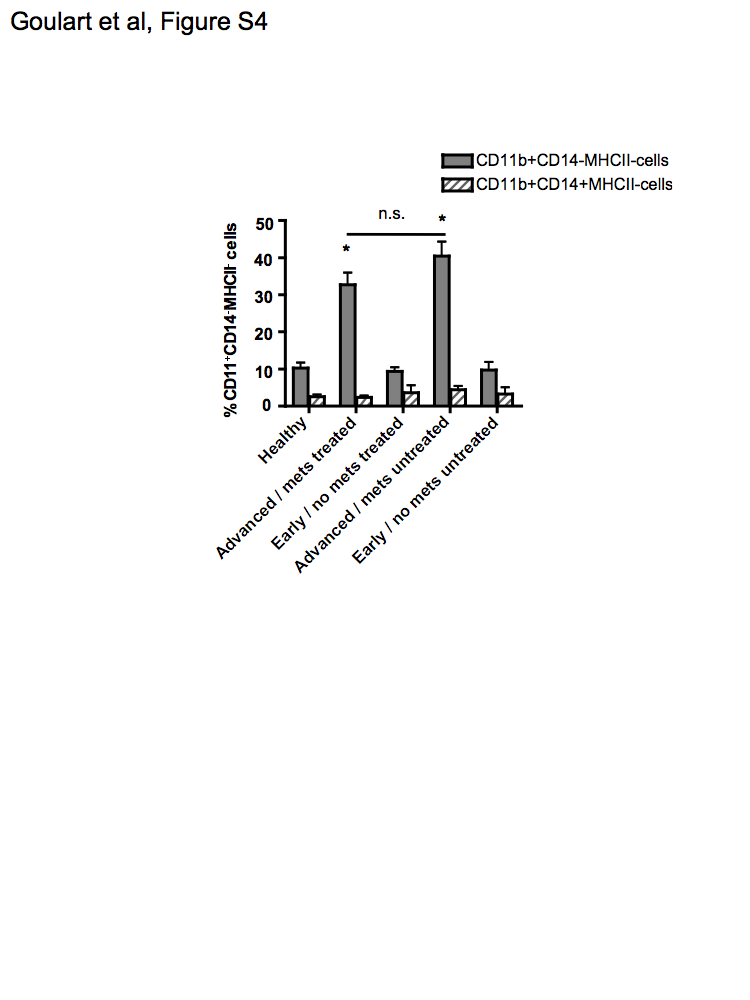

Supplement: Figure S4 — No significant effect of pretreatment on MDSC burden. Analysis of the average CD11b+CD14−MHCII− population frequency in treated (n = 17) or untreated dogs with advanced stage or metastatic tumors (n = 13) compared to control dogs (n = 18). There was a significantly higher percentage of CD11b+CD14−MHCII− cells in dogs with advanced cancer treated or untreated compared to healthy dogs (32.69±3.24%, 40.42±3.86% vs. 10.24±1.412%, respectively). N.S., not statistically significant (there was no significant difference between samples that had been treated compared to those from untreated samples). Mean ± SEM are shown (* indicates P<0.0001). (TIF) [file pone.0033274.s004.tif]
