# Supplementary material for: Identification of Myeloid Derived Suppressor Cells in Dogs with Naturally Occurring Cancer
Source: PLoS One. 2012 Mar 13;7(3):e33274. doi: 10.1371/journal.pone.0033274 (PMC3302813; doi:10.1371/journal.pone.0033274)
Supplement: Table S1 — Summary data for dogs with advanced stage or metastatic tumors. (DOC) [file pone.0033274.s005.doc]

Goulart et al, Table S1

**Table S1. Summary data for dogs with advanced stage or metastatic tumors**

|  |  |  |  |  |  | **% of CD11^+^** | **% of CD11^+^** |  |
| --- | --- | --- | --- | --- | --- | --- | --- | --- |
|  |  |  |  |  |  | **CD14^-^** | **CD14^+^** |  |
|  | **Sample** | **Age / Gender / Breed** | **Tumor Type** | **Tumor Characteristics** | **Metastasis** | **MHCII^-^** | **MHCII^-^** | **Treatment** |
| 1 | fresh | 7y FS Labrador Retriever | OSA | Necrosis and venous tumor embolus, | No | 29.6 | 0.94 | NSAID, T |
|  |  |  |  | High grade |  |  |  |  |
| 2 | fresh | 8y FS Newfoundland | OSA | Lytic bone lesion | No | 39 | 2.13 | NSAID, T |
| 3 | frozen | 10y FS Greyhound | OSA Ŧ | Lytic bone lesion | Yes. LG | 19.1 | 5.66 | Su, NSAID |
| 4 | frozen | 7y MN Lab Retriever | OSA | Lytic bone lesion | No | 11.7 | 2.99 | NSAID, T |
| 5 | fresh | 4y FS Mixed Breed | OSA | Ribs masses and pleural effusion | Yes. LG | 50.5 | 7.26 | NSAID, T |
| 6 | fresh | 4y MN Mixed Breed | OSA | Mixed lytic proliferative bone lesion | No | 39.9 | 1.51 | NSAID, T |
| 7 | frozen | 6y MN Greyhound | OSA | Mixed lytic proliferative bone lesion | No | 20.6 | 0.99 | NSAID, T |
| 8 | fresh | 11y MN Golden Retriever | OSA | Mixed lytic proliferative bone lesion, | Yes. LN | 22 | 2.25 | T |
|  |  |  |  | severe destruction |  |  |  |  |
| 9 | fresh | 13y FS Lab. Retriever | HSA | Skeletal, lytic bone lesion, fracture | No | 52,4 | 0.46 | No |
| 10 | frozen | 12y MN Border Collie | HSA | Oral | Yes. L, BL | 39.3 | 2.25 | No |
| 11 | frozen | 8y MN Lab. Retriever | HSA Ŧ | Skeletal, large edema, | Yes. L, LG,S | 41.7 | 1.98 | Su |
|  |  |  |  | serosanguineous. fluid |  |  |  |  |
| 12 | frozen | 10y FS Boxer | HSA Ŧ | Splenic nodules | Yes. L | 51.9 | 2.45 | Su, NSAID |
| 13 | frozen | 9y MN Mixed Breed | HSA Ŧ | Splenic, mast cell tumor | Yes. L, M | 13.1 | 3.77 | Su, CH (D,C) |
| 14 | fresh | 10y MN Golden Retriever | HSA | Splenic multiple nodules | No | 62 | 10.1 | No |
| 15 | frozen | 11y MI Rat Terrier | SCS Ŧ | Masses on axillary region/scapula and chest, | Yes | 42.1 | 1.02 | Su |
|  |  |  |  | rapid regrowth |  |  |  |  |
| 16 | frozen | 8y MN Bernese Mountain | Hist.Sarc Ŧ | Lung nodules | Yes. LG | 52.8 | 1.36 | NSAID, CH |
|  |  | dog |  |  |  |  |  | (D, C, Lo) |
| 17 | frozen | 2y MI Golden Retriever | Hist.Sarc | Tumor extent from orbit through hard palate | possibly | 30.9 | 1.31 | No |
| 18 | frozen | 12y FS Lab. Retriever | Soft T.Sarc | Multiple masses | Yes | 20.3 | 8.96 | No |
| 19 | fresh | 10y MN Border Collie | SCC | Tonsillar, large neck mass | Yes. N | 39.9 | 4.36 | No |
| 20 | fresh | 10y MN Scottish Terrier | AC | Prostatic, TCC bladder | Yes. L | 19.9 | 1.52 | NSAID |
| 21 | fresh | 7y MN Bull Mastiff | AC | Thyroid, large mass on left side, | No | 32.9 | 4.3 | No |
|  |  |  |  | small on right side |  |  |  |  |
| 22 | fresh | 13y MN Mixed Breed | AC | Hepatocellular (large mass), | No | 29.1 | 0.82 | No |
|  |  |  |  | bladder leiomyoma |  |  |  |  |
| 23 | frozen | 9y MN English Spring | AC | Anal sac, lymphadenopathy, | Yes. LN, LG | 40 | 4.64 | NSAID |
|  |  | Spaniel |  | hypercalcemia of malignancy |  |  |  |  |
| 24 | fresh | 5y MN Great Dane | AC | Thyroid, rapid growth with invasion, | Yes. B | 65.9 | 9.26 | No |
|  |  |  |  | destruction of underlying bone |  |  |  |  |
| 25 | frozen | 7y FS Dalmatian | AC | Mammary, multiple nodules | No | 34.4 | 7.63 | No |
| 26 | frozen | 10y FS Rottweiler | MCT | Iliac and inguinal lymph node nodules | Yes. LN | 29.1 | 1.21 | AH |
| 27 | frozen | 10y FS Lab. Retriever | MCT | Multiple nodes | Yes | 32.8 | 0.6 | AH |
| 28 | frozen | 7y FS Boxer | MCT | Multiple nodes | Yes | 38.2 | 0.6 | No |
| 29 | fresh | 9y FS German W. Pointer | MCT | Multiple nodes | Yes | 53.1 | 2.42 | No |
| 30 | fresh | 10y FS Lab. Retriever | MCT | Multiple tumor | Yes. LN | 27.1 | 0.47 | No |

Ŧ Recurrence after incompletely excised FS, female spayed; MN, male neutered; MI, male intact; OSA, osteosarcoma; HSA, hemangiosarcoma; SCS, spindle cell sarcoma; Hist. Sarc, histiocytic sarcoma; Soft T.Sarc, soft tissue sarcoma; SCC, spindle cell carcinoma; AC, adenocarcinoma; TCC, transitional cell carcinoma; L, liver; LG, lung; BL, bladder; S, spleen; M, mesentery; B, bone; NSAID, non-steroidal anti-inflammatory drug; T, tramadol; Su, surgery; CH, chemotherapy; D, doxorubicin; C, cyclophosphamide; Lo, lomustine; AH, antihistamine
