# Supplementary material for: Identification of Myeloid Derived Suppressor Cells in Dogs with Naturally Occurring Cancer
Source: PLoS One. 2012 Mar 13;7(3):e33274. doi: 10.1371/journal.pone.0033274 (PMC3302813; doi:10.1371/journal.pone.0033274)
Supplement: Table S2 — Summary data for dogs with early stage non-metastatic tumors. (DOC) [file pone.0033274.s006.doc]

Goulart et al, Table S2

**Table S2. Summary Data for Dogs with Early Stage non-metastatic tumors**

|  |  | |  |  | |  | |  | |  | **% of CD11b^+^** | **% of CD11b^+^** |  |
| --- | --- | --- | --- | --- | --- | --- | --- | --- | --- | --- | --- | --- | --- |
|  |  |  | |  |  | |  | | |  | **CD14^-^** | **CD14^+^** |  |
|  | **Sample** | **Age / Gender /Breed** | | **Tumor type** | **Tumor Characteristics** | | | | **Metastasis** | | **MHCII^-^** | **MHCII^-^** | **Treatment** |
| 1 | fresh | 10y FS Labrador Retriever | | Osteosarcoma | Moderate bone lysis | | | | No | | 5.77 | 2.7 | NSAID, T |
|  |  |  | |  | distal radius/ulna | | | |  | |  |  |  |
| 2 | frozen | 11y FS Labrador Retriever | | Spindle Cell Sarcoma Ŧ | Small masses on the neck | | | | No | | 13 | 25 | CST, T |
| 3 | frozen | 8y FS Beagle | | Spindle Cell Sarcoma | Carpal pad low grade tumor | | | | No | | 7.38 | 0.92 | NSAID |
| 4 | frozen | 14y MN Mixed Breed | | Soft Tissue Sarcoma Ŧ | Low grade tumor | | | | No | | 14.4 | 0.73 | Su, R, Cs, P |
|  |  |  | |  | dorsal lumbar region | | | |  | |  |  |  |
| 5 | frozen | 9y MN Labrador Retriever | | Histiocytic Sarcoma Ŧ | Small mass on left hock | | | | No | | 12.5 | 6.72 | No |
| 6 | frozen | 8y MN Labrador Retriever | | Spindle Cell Sarcoma | Small mass on left hindlimb | | | | No | | 5.83 | 1.3 | NSAID, T |
| 7 | fresh | 11y FS Chow Chow | | Spindle Cell Carcinoma | Mass on nasal planum | | | | No | | 13.2 | 1.37 | NSAID, T |
| 8 | fresh | 13y MN Miniature Poodle | | Transitional Cell | Bladder | | | | No | | 5.36 | 1.04 | NSAID |
|  |  |  | | Carcinoma |  | | | |  | |  |  |  |
| 9 | frozen | 8y FS West Highland White terrier | | Transitional Cell | Bladder, early stage, | | | | No | | 5.57 | 1.1 | No |
|  |  |  | | Carcinoma | low grade | | | |  | |  |  |  |
| 10 | fresh | 12y MN German Shepherd | | Adenocarcinoma | Thyroid mass | | | | No | | 11.1 | 1.9 | No |
| 11 | frozen | 12y FS Beagle | | Adenocarcinoma | Hepatocellular | | | | No | | 14.4 | 3.37 | CST, P |
| 12 | fresh | 12y MN Labrador Retriever | | Squamous Cell | Small mass on nasal planum | | | | No | | 8.63 | 2.87 | NSAID, Ca |
|  |  |  | | Carcinoma |  | | | |  | |  |  |  |
| 13 | frozen | 10y FS Gordon Setter | | Transitional Cell | Bladder | | | | No | | 5.03 | 0.24 | NSAID |
|  |  |  | | Carcinoma |  | | | |  | |  |  |  |
| 14 | fresh | 7y MN Weimaraner | | Mast Cell Tumor Ŧ | Grade II | | | | No | | 12.6 | 2.86 | Su |
| 15 | frozen | 8y FS Rhodesian Ridgeback | | Mast Cell Tumor Ŧ | Grade II | | | | No | | 6.25 | 1.05 | Su, P |

Ŧ Recurrence after incompletely excised tumor FS, female spayed; MN, male neutered; NSAID, non-steroidal anti-inflammatory drug; T, tramadol; Su, surgery; CST, corticosteroid; R; radiation; D, doxorubicin; Ca, carboplatin. P, Palladia
