# Supplementary material for: Identification of Myeloid Derived Suppressor Cells in Dogs with Naturally Occurring Cancer
Source: PLoS One. 2012 Mar 13;7(3):e33274. doi: 10.1371/journal.pone.0033274 (PMC3302813; doi:10.1371/journal.pone.0033274)
Supplement: Table S3 — Table of cancer patient samples and the experiment in which the PBMCs were used. (DOC) [file pone.0033274.s007.doc]

Goulart et al, Table S3

**Table S3. Table of cancer patient samples and the experiment in which the PBMCs were used.**

| Sample Number | Experiment (s) | Figure (s) |
| --- | --- | --- |
| Sample 3 - Healthy | Immunophenotyping | 1A |
| Sample 5 - Advanced Stage | Tcell proliferation, cytospin | N.S |
| Sample 5 - Healthy | Tcell proliferation | N.S |
| Sample 6 - Advanced Stage | Tcell proliferation, RT-PCR | 4, 5 |
| Sample 6 - Healthy | Tcell proliferation, RT-PCR | 4, 5 |
| Sample 7 - Early Stage | Tcell proliferation, cytokine elaboration, | N.S |
|  | cytospin |  |
| Sample 8 - Advanced Stage | Tcell proliferation, cytokine elaboration, | 3C,3D |
| Sample 8 - Early Stage | Tcell proliferation, cytokine elaboration | N.S |
| Sample 9 - Early Stage | Immunophenotyping | 1A |
| Sample 10 - Healthy | Tcell proliferation, cytokine elaboration | N.S |
| Sample 10 - Advanced Stage | RT-PCR | N.S |
| Sample 11 - Advanced Stage | Immunophenotyping | 1A |
| Sample 12 - Healthy | Tcell proliferation, cytokine elaboration | 3C,3D |
| Sample 13 - Healthy | RT-PCR, Tcell proliferation, cytokine | N.S |
|  | elaboration |  |
| Sample 14 - Advanced Stage | RT- PCR | N.S |
| Sample 16 - Advanced Stage | RT- PCR | N.S |
| Sample 17 - Healthy | RT-PCR | N.S |
| Sample 18 - Advanced Stage | Immunophenotyping | S1 |
| Sample 18 - Healthy | Tcell proliferation, cytokine elaboration | N.S |
| Sample 19 - Advanced Stage | Tcell proliferation | 3A |
| Sample 20 - Advanced Stage | Tcell proliferation | 3B |
| Sample 22 - Advanced Stage | Cytospin | 1B |
| Sample 23 - Advanced Stage | RT- PCR | N.S |
| Sample 29 - Advanced Stage | RT-PCR,cytospin | N.S |
| Sample 30 - Advanced Stage | Tcell proliferation, cytokine elaboration | N.S |
|  | cytospin |  |

N.S = not shown
