# Supplementary material for: Identification of Myeloid Derived Suppressor Cells in Dogs with Naturally Occurring Cancer
Source: PLoS One. 2012 Mar 13;7(3):e33274. doi: 10.1371/journal.pone.0033274 (PMC3302813; doi:10.1371/journal.pone.0033274)
Supplement: Table S4 — Primer sequences for genes evaluated by semi-quantitative PCR. (DOC) [file pone.0033274.s008.doc]

Goulart et al, Table S4

**Table S4. Primer sequences for genes evaluated by semi-quantitative PCR**

| **Gene** |  | **Primer Sequence (5'-3')** | **Size** |
| --- | --- | --- | --- |
| IL-10 | Forward | GTCCCTGCTGGAGGACTTTAAGA | 443 bp |
|  | Reverse | TGGTCGGCTCTCCTACATCTCG |  |
| ARG-1 | Forward | TGGCCTGCTGGAGAAACTTA | 190 bp |
|  | Reverse | CAGCACCAGGCTAGTCCTTC |  |
| INOS-2 | Forward | AAGTCCAAGTCTTGTCTGGGAGC | 185 bp |
|  | Reverse | TCCTTTGTTACTGCTTCCACCCT |  |
| TGF-β | Forward | AGTTAAAAGCGGAGCAGCATGTGG | 434 bp |
|  | Reverse | GATCCTTGCGGAAGTCAATGTAGAGC |  |
| β-actin | Forward | CCAGCAAGGATGAAGATCAAG | 100 bp |
|  | Reverse | TCTGCTGGAAGGTGGACAG |  |
